# Free radial forearm adiposo-fascial flap for inferior maxillectomy defect reconstruction

**DOI:** 10.4103/0970-0358.53018

**Published:** 2009

**Authors:** Krishnakumar Thankappan, Nirav P. Trivedi, Mohit Sharma, Moni A. Kuriakose, Subramania Iyer

**Affiliations:** Department of Head and Neck Surgery, Amrita Institute of Medical Sciences, Kochi, India

**Keywords:** Fascia only flap, Free radial forearm flap, Maxillary reconstruction

## Abstract

A free radial forearm fascial flap has been described for intraoral reconstruction. Adiposo-fascial flap harvesting involves few technical modifications from the conventional radial forearm fascio-cutaneous free flap harvesting. We report a case of inferior maxillectomy defect reconstruction in a 42-year-old male with a free radial forearm adiposo-fascial flap with good aesthetic and functional outcome with minimal primary and donor site morbidity. The technique of raising the flap and closing the donor site needs to be meticulous in order to achieve good cosmetic and functional outcome.

## INTRODUCTION

A free radial forearm fascial flap has been described for intraoral reconstruction.[1,2] An adiposo-fascial radial forearm flap when used in intraoral reconstructions is not riddled with he problems of hair growth, forms an effective oro-nasal and/or oro-antral seal and at the same time avoids a skin graft on the very visible donor site. We report a case of inferior maxillectomy defect reconstruction with free radial forearm adiposo-fascial flap with good aesthetic and functional outcome with minimal primary and donor site morbidity.

## CASE REPORT

**Figure 1:**
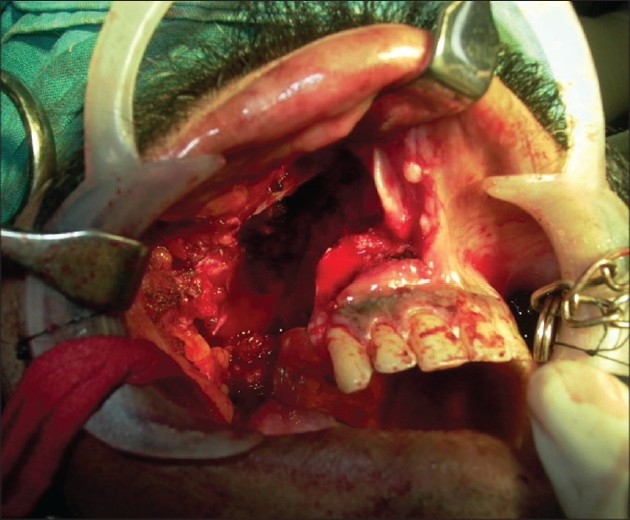
Inferior maxillectomy defect

A 42-year-old male presented with a history of swelling on the right side of the upper jaw in the molar area. A biopsy proved it to be low-grade mucoepidermoid carcinoma. An inferior maxillectomy with resection of maxillary alveolus distal to the canine tooth and adjacent hard palate was carried out. This resulted in a Class IIa defect according to classification of maxillectomy defects by Brown *et al.*[3][Figure 1]. The defect size was 3 cm × 4cm. The objective of reconstruction was to get an oro-antral separation and to provide a base for the dental prosthesis. The defect was reconstructed with a free radial forearm adiposo-fascial flap harvested from the left forearm.

**Figure 2:**
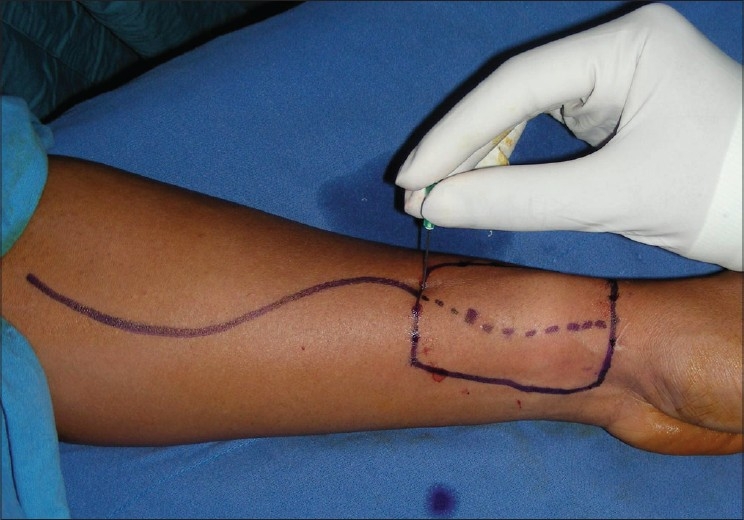
Flap marking with inked needle

**Figure 3:**
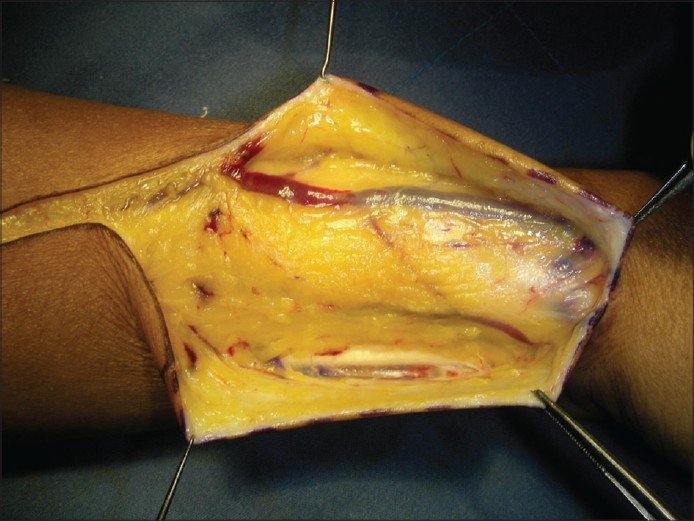
Suprafascially-raised skin, a layer of fat retained on the fascia

**Figure 4:**
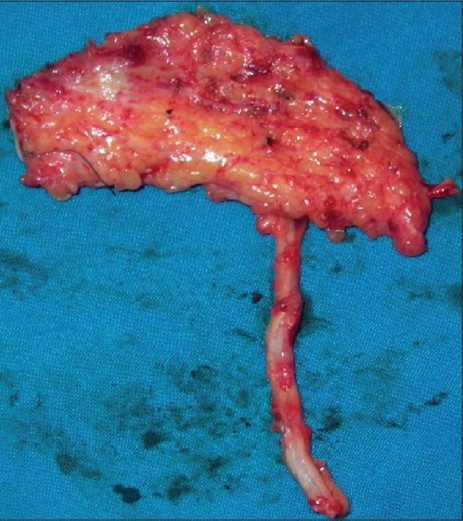
Adiposo-fascial flap with the pedicle

Adiposo-fascial flap harvesting involves few technical modifications from the conventional radial forearm fascio-cutaneous free flap harvesting. The flap was marked on the forearm skin. The margin of the flap was marked with a hypodermic needle dipped in methylene blue, the tip of the needle needed to reach the subcutaneous layer [Figure 2]. A lazy “S” incision was placed extending from a point 2 cm proximal to the wrist crease to a point 2 cm distal to the ante-cubital fossa. The skin flap was elevated in the subcutaneous plane to leave the layer of adipose tissue on the fascia [Figure 3]. The fascia with fat, incised along the dye markings, was harvested based on the radial artery [Figure 4]. The methylene blue marking helped to limit the dissection beyond the requirement. The width of the flap that can be harvested by this technique is similar to the conventional radial forearm flap. The skin over the flap site is raised at a superficial level to expose the adiposofacial tissue. Hence, large flaps may compromise the vascularity of this skin flap and lead to necrosis at the incision site. So it would be safer to restrict the size of the flaps to the width of the forearm. The donor site was closed primarily in two layers, the subcutaneous tissue with absorbable 3-0 Polyglactin 910 (Vicryl^®^) and skin with 3-0 nylon suture. The two layered closure allowed better approximation of the skin edges. A suction drain was used to prevent any subcutaneous hematoma. This was removed after 3 days. The donor site required no immobilization. Immobilization is usually required for 1 week after the harvest of the conventional fasciocutaneous flap and split skin graft for the defect. The flap was then sutured to the defect edges and the radial artery was anastomosed to the superior thyroid artery and the accompanying venae commitantes was anastomosed to a direct tributary of the internal jugular vein through a right neck crease incision. The flap was monitored by the color, by observing the nature of bleeding from the adiposal tissue by pinprick method, and from the Doppler signals of the artery. The patient was kept on nasogastric tube feeds for 5 days; after 5 days, a soft diet was started. Solid food was allowed after 2 weeks.

**Figure 5:**
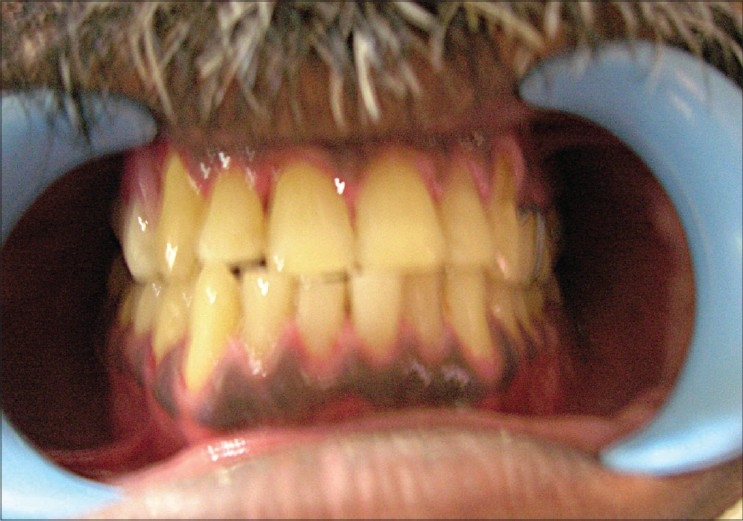
Dental rehabilitation with removable dentures

**Figure 6:**
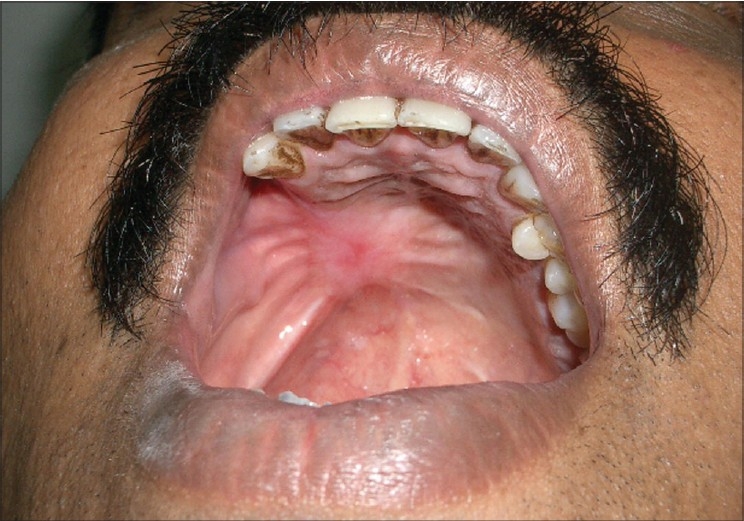
Reconstruction outcome after 2 years of follow-up

The primary site healed well with rapid re-epithelialization of the fascial subcutaneous layer to achieve a mucosal surface. The flap underwent some amount of contraction providing a taut immobile base for the prosthetic dental rehabilitation [Figure 5]. There was no restriction of mouth opening before or after the surgery. The defect did not crossover to the cheek, hence, there was no significant obliteration of the sulcus. It was limited laterally to the bony alveolus. This prevented the cheek from retracting in. Since this was a Stage II disease, the patient did not receive any adjuvant treatment. Figure 6 shows a view of the palate well mucosalized with no intraoral hair at the end of 2 year follow-up. Functionally, he could eat a normal diet, his speech was normal, and he had no nasal regurgitation. The donor site healed well. Primary and donor sites were aesthetically satisfactory.

## DISCUSSION

A free radial forearm flap is the workhorse flap in oral cavity reconstruction.[4] It has been used for maxillectomy reconstruction with satisfactory results.[5] Donor site morbidity in a radial forearm flap is troublesome[6] and morbidity due to hair growth in oral cavity is also common. Various techniques have been proposed to minimize the donor site morbidity.[7,8] Free radial forearm fascial flap has been described for intraoral reconstruction.[1,2] Pre-lamination of fascial radial forearm flap with autologous mucosa[9] and tissue engineered mucosa[10] has been reported. Poeschel, *et al.*[11] compares pre-laminated and non-prelaminated radial forearm flaps for oral cavity reconstruction. Early wound healing difficulties are seen more often in the pre-laminated flap group, whereas donor site problems occurred more frequently in the non-prelaminated group. In a study of 35 patients comparing pre-laminated fascio-mucosal flap and the fasciocutaneous radial forearm flap for intraoral reconstruction, Nehrer – Tairych, *et al.*[12] conclude that using the pre-laminated fascio-mucosal radial forearm flap minimizes the donor-site morbidity.

There are reports suggesting early mucosalization of fascial flaps in the oral cavity.[13] It is therefore possible to use the adiposo-fascial flap without pre-lamination. The mechanism of re-epithelialization in muscle-only flaps in intraoral reconstruction is described.[14] In comparison to the fascio-cutaneous flap, the adiposo-fascial flap undergoes a certain extent of scar contraction. This can be an advantage in reconstructing the palatal defect. The taut fascial flap offers support for dental rehabilitation. However, this flap may not be suitable for defects extending to the soft palate as it may result in contraction and velo-pharyngeal incompetence.

Class 2 defects[3] produce oroantral or oronasal fistula. The main aim of reconstruction here is to achieve this seal. A class 2 defect can be successfully obturated in a dentate maxilla. If a flap reconstruction is chosen for the defect, the height of the antrum does not require reconstruction allowing a choice of local, pedicled, or free flaps. Smaller defects involving the alveolar ridge, teeth, and surrounding mucosa can be covered with a local flap. Of the many local flaps described, the palatal flap[15] is reliable. A temporalis flap[16] is the next option. The disadvantages include the possibility of trismus and donor site deformity. In elderly patients, the risk of a long procedure under general anesthesia is high and females usually have an acceptable cosmetic temporal deformity as compared with males. A free radial forearm flap is the other choice. We consider it the flap of choice for these defects in young healthy individuals.

We report a case of inferior maxillectomy defect following resection for a low-grade mucoepidermoid carcinoma reconstructed with free fascial forearm flap with minimal primary and donor site morbidity. No pre-lamination method was performed in this case. The new mucosalized area looked similar to the other areas of the oral cavity and the donor site needed skin grafting. Though our patient did not receive any adjuvant treatment, it would be noteworthy to mention that this type of modification in the flap design does not contraindicate adjuvant radiotherapy.
